# Relationship of litterfall anomalies with climatic anomalies in a mangrove swamp of the Yucatan Peninsula, Mexico

**DOI:** 10.1371/journal.pone.0307376

**Published:** 2024-08-28

**Authors:** Claudia Teutli-Hernández, M. Fernanda Cepeda-González, Jorge L. Montero-Muñoz, Israel Medina-Gómez, Rosa María Román-Cuesta, Jorge A. Herrera-Silveira

**Affiliations:** 1 Universidad Nacional Autónoma de México, Escuela Nacional de Estudios Superiores Unidad Mérida, Mérida, México; 2 Centro de Investigación y de Estudios Avanzados del IPN, Unidad Mérida, Mérida, México; 3 European Commission, Joint Research Centre, Forests & Bioeconomy Unit, Brussels, Belgium; 4 Laboratorio Nacional de Resiliencia Costera, Mérida, México; Western Carolina University, UNITED STATES OF AMERICA

## Abstract

Among the set of phenological traits featuring mangrove ecosystems, litterfall production stands out with marked intra-annual and longer-term variation. Furthermore, mangrove forests resilience is one of the most important ecological attribute, reconciling the juxtaposed terrestrial and marine environment such transitional systems occupy. However, world’s mangroves are nowadays facing recurrent climatic events, reflected in anomalies depicted by major drivers, including temperature and precipitation. This physical-environmental setting may either constrain or favor overall forest productivity. A combination of time series analysis (spectral density and cross-correlation techniques) and statistical model fitting (General additive model) was implemented to explore trends in total litterfall of a well-developed mangrove forest in southeastern Gulf of Mexico (Celestun Lagoon, SE Mexico) and potential association with the varying behavior of temperature (°C) and precipitation (mm month^-1^), highlighting their anomalies. The results are consistent with a synchronous response between litterfall production and climatic variables (mean monthly temperature and total monthly precipitation). Concurrent peak litterfall production in Celestun lagoon with high temperatures and precipitation occurred during June and October, featuring a two-month time lag for the response time. More than half of the litterfall anomalies (53.5%) could be reflecting either multiple sources of climatic anomalies (maximum, minimum, and monthly average temperature and monthly total precipitation) or single point events (cyclone landfall). This relationship dynamics showed an interannual persistence (1999–2010). The structure portrayed by the litterfall time-series was not unequivocally related to climatic anomalies. Arguably, climatic anomalies behave with different intensities and even may exhibit complex interactions among them. The study of anomalies provides a baseline for a better grasp of: i) mangrove anomalies responses and ii) their vulnerability to these extremes.

## Introduction

As successive extreme events unfolded during the last decade, ranging from persistent heat waves, enhanced tropical cyclones frequency, and drought periods lasting longer [1–3], the projected scenarios suggest that pressing ecological disturbances over wetland ecosystems may lead to structure and function alterations that potentially compromise the ecological balance among species, including changes in their relative abundance across the landscape and community composition [4, 5].

The increased recurrence prognosis for either stand-alone or concatenated events suggests the threat for vast tropical wetland areas and their multiple ecological services will intensify. Mangrove forests stand out from these transitional zones, depicting high ecological stability and supporting high productivity levels despite enduring a continuing fluctuating physical-environmental background.

Mangrove canopy regrowth may occur following a hurricane landfall, although the pre-storm height levels achievement is constrained by tree size. This relationship may select shorter mangrove stands, which attain relatively higher resilience than taller forest ecotypes (e.g., riverine mangroves) [6]. A sharp increase in storms frequency and intensity might entail less between-events adapting time for exposed ecosystems. This variation in the disturbance regime may potentially drive both morphologic and size alterations in these ecotones or, ecological shifting into marshes, mudflats, or open water systems [7].

The extent of hurricane damage over mangrove forests is usually site-specific and a function of wind speed, trajectory, local topography, and as aforementioned, mangrove type (tallest trees undergoing the worst damage) [8, 9]. The impacts experienced by individual trees includes defoliation, branches detachment, uprooting, and further senescence of flooded roots [10]. From the landscape point of view, the spatial pattern left by stormy winds and heavy rainfall brought about by hurricanes contributes to a legacy in impacted vegetated areas. This disturbances history may set further recovery trajectories in the aftermath of such events [11]. Some mangrove species may avoid severe damage from extreme events either by mechanically resisting the wind-induced stems breaking and tree uprooting or, depicting some level of species-specific physiological plasticity after breaking via resprouting and accommodating natural regeneration [12, 13].

The litterfall constitutes as well a compounded ecosystem response, since it may aggregate leaves, branches, flowers, fruits, and a miscellaneous collection of structures. Consequently, the varying litterfall biomass and productivity among constituents is often considered associated with forest productivity. These patterns may mask internal synergies and feedbacks between reproductive and structural constituents and taper off the otherwise abrupt phenological responses of sensitive mangrove forests to local climatologic traits, as precipitation, temperature, evaporation, and freshwater input.

The soil accumulation in mangrove ecosystems, ascribed both to their high productivity along with local hydrological conditions and flushing characteristics, renders these forests a high resilience. Moreover, the ensuing inundation regime across mangroves underlies the particular physical-environmental setting that further modulates litterfall dynamics. Future changes in tropical cyclone activity and easterly waves [14] have the potential to exacerbate ongoing processes faced by mangroves, as sea-level rise and stormy weather episodes [15].

The Yucatan Peninsula, in the southeastern Mexico, has been historically subject to the impact of tropical storms and hurricanes of varying intensity and magnitude. Short-term studies indicate mangrove productivity is strongly related to the wet/dry seasonal variability [16]. However, the lack of long-term datasets documenting mangrove responses for a number of plant traits, including litterfall production and seasonality, prevents an adequate understanding of non-seasonal behavior. The environmental stress elicit forest growth and developmental patterns in tropical latitudes. As aforementioned, relevant forest functions including understory microclimate regulation [17], nutrients outwelling and the coupling extent with adjacent water bodies [18], as well as Carbon storage capabilities of mangrove wetlands are sensitive to litterfall dynamics [19]. Therefore, the assessment of time-dependent litterfall production dynamics could help addressing these forests sensitivity to the environmental matrix, in terms of their responses to climatic anomalies and extreme events.

This paper tackled two aspects: 1) address a description of the presence of daily and monthly anomalies in the precipitation and temperature, taking the period within 1961 and 2020 as the basis to identify the seasonal pattern of said variables. 2) Assess the influence of precipitation and temperature anomalies on litterfall productivity, focused in the period within 1999–2010 in Laguna de Celestún (SE Mexico).

The working hypothesis of this study points out the litterfall production seasonality is altered in the aftermath of extreme hydrometeorological events crossing the region. The high defoliation experienced under stormy weather sets lower litterfall production for the subsequent year when a hurricane made landfall nearby the forest. This relationship would be reflected in abrupt litterfall fluctuations and further stabilization as a function of time.

Implications tied to future cyclonic activity and eastern waves acting upon vast tropical regions covered by mangroves indicate that both seasonal rainfall and extreme precipitation during hydrometeorological events will impact the hydrologic cycle across these forest in the Yucatan [17]. This nuanced knowledge provides a relevant reference to understand further litterfall responses to hydrometeorological events, allows envisioning the impact of climatologic changes in this phenology dynamics and helps prioritizing areas for restoration efforts if needed.

## Materials and methods

### Study area

**Fig 1:**
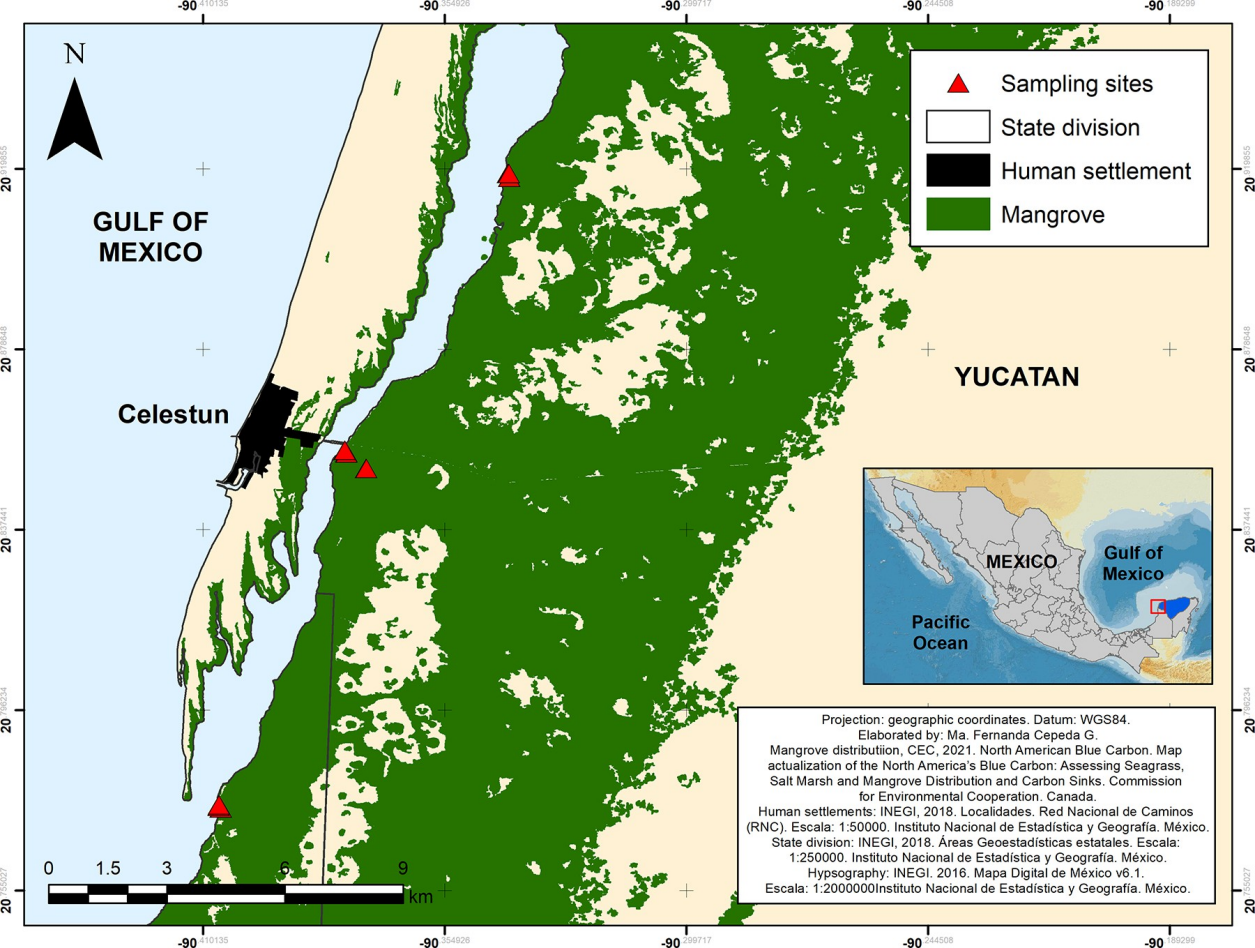
Location of litterfall sampling sites in the Celestun lagoon, Yucatan, Mexico. Metadata: CEC basic metadata (https://www.arcgis.com/sharing/rest/content/items/54d358afe94e467caada184f9c2b16df/info/metadata/metadata.xml?format=default&output=html); state division (http://geoportal.conabio.gob.mx/metadatos/doc/html/dest2018gw.html); human settlements (http://geoportal.conabio.gob.mx/metadatos/doc/html/lcineg18gw.html); hypsography (https://www.inegi.org.mx/app/biblioteca/ficha.html?upc = 889463604389).

The “Ria Celestun Biosphere Reserve”, located in the Yucatan Peninsula, SE Mexico (Fig 1), is characterized by geohydrological karst geomorphology with enhanced permeability, lacking of surface rivers or streams. The entire coastal ecosystem receives freshwater from groundwater controlled by meteoric recharge [20], both locally and regionally. Thus, an important geohydrological feature is the underground freshwater discharges into the Celestun coastal lagoon, mainly in the upper (north) and mid-lagoon zones, occurring both through diffuse and point source springs [20]. Groundwater can mobilize from remote areas, several kilometers inland across the southern karstified Yucatan platform [21]. From a landscape view, the rainfall recharge reconciles both local and region-scale influences over the coastal area.

This area experiences hydroclimatic instabilities under the influence of intra- and inter-annual variability of rainfall, winds, and temperature. The driest season (March-May) is characterized by high temperature (32 to 42˚C) and enhanced evaporation rates, the rainy season (June-October) depicts large precipitation (140 to 250 mm/month) and diminished salinity (surface and interstitial water). Towards the end of the year (November-February) intense winds (˃60 km/h) and sudden drop in air temperature (31 to 8˚C) associated with polar frontal systems traversing the Gulf of Mexico characterizes the "nortes" season (northwinds). The “nortes” are sometimes accompanied by slight rainfall (10–60 mm / month).

The aforementioned geohydrological, climatic and geomorphologic setting (one-single inlet and a relatively narrow tidal channel controlling the lagoon flushing characteristics [22]) governs a hydrological-based zonation prevailing along the coastal lagoon: a mesohaline inner system with 5–12 salinity and high nitrate (>40 μM) and silicate (>300 μM) reflecting groundwater discharges venting into the inner-most lagoon; a mid-lagoon characterized by 15–30 salinity and phosphate concentration within 0.5–2.0 μM; and the southern inlet area, depicting relatively low nutrient concentrations (nitrate <10 μM and silicate <25 μM) and evident marine influence (salinity >30) which may extend farther into the lagoon across the entrance channel under a varying wind forcing.

The Celestun lagoon mangrove ecosystem consists of red mangrove *Rhizophora mangle*, white mangrove *Laguncularia racemosa* (both dominating species along the fringe forest), black mangrove *Avicennia germinans* (prevalent as a basin forest), and button mangrove *Conocarpus erectus* (less represented in the area and only occupying the dwarf forest) [17, 23]. Sampled sites for the current study include all ecotypes of mangroves present in the area.

### Litterfall sampling

The litterfall dataset span eleven years (1999–2010) of monthly samplings over two 10x10 m plots with five 0.25 m^2^ traps each, across the three prior mangrove zones (n = 30). The litterfall collectors (traps) were positioned ~1.3 m above the highest tide. Back in the lab, litterfall content was dried at 75°C for an average of 72 hours, weighed (g.d.w.m^-2^ day^-1^), sorted into “leaf” and “non-leaf” and then classified by species and constituents (fruits and flowers, branches, and other elements). The total monthly litterfall per day in g.d.w.m^-2^ was used as dependent variable–sum of all the components of all the species [24].

### Climate information

Climatic data were provided by the National Water Commission [25]. It includes: daily and monthly maximum, minimum and average temperature (°C), and daily and total monthly precipitation (mm) between 1961 and 2020 (meteorological station of Celestun). Notwithstanding the potential remote provenance of groundwater flowing towards the Celestun mangrove swamp, the rather unavailable information on water discharges for this area lead us to limit this study to rainfall recorded through the aforementioned local meteorological station.

The hurricanes incidence information was obtained from public archives by NOAA (IBTrACS https://www.ncdc.noaa.gov/ibtracs/) [26] between 1961 and 2020. For such purpose, a 40 km buffer was created throughout the Celestun Reserve polygon and the cyclones intersecting the buffer were systematically detected. Besides, maximum sustained wind speed at the closest point to the Reserve was recorded.

Since there are hydrometeorologic events bringing about heavy rainfall which surface extension influence is wider than 40 km, the IBTrACS information was added with precipitation recordings (accumulated precipitation during each event in mm) for cyclones making landfall nearby Celestun, beyond that of the designated buffer [27]. Thus, both cyclones categories affecting the buffer and those farther were related to single point (daily) and persistent (monthly) precipitation anomalies.

### Statistical analysis

Since this study aims tackling the potential relationship between litterfall dynamics and atypical climatic events, a dual statistical approach was implemented, each defined by a specific timeframe: 1) a full description of precipitation and temperature anomalies allows identifying both persistence and recurrence of daily events and the period within 1961–2020 was deemed suitable to unveil the seasonal pattern enclosed by these variables. 2) The time-dependent litterfall behavior under the influence of both precipitation and temperature anomalies was constrained by the available litterfall data and consequently, the assessment was focused within the interval of 1999–2010.

Moreover, time series of total precipitation (mm) and average minimum and maximum temperature (°C) comprising the period within 1961–2020, with daily and monthly sampling frequency were analyzed. Additionally, the total litterfall productivity (g.d.w.m^-2^ day^-1^) time series encompasses 1999–2010 with a monthly sampling resolution.

To describe the monthly variation of litterfall, total precipitation, and mean temperature, a graph of 95% confidence intervals was obtained for the period between 1999 and 2010.

A cross-correlation analysis allowed detecting the litterfall response to mean temperature and total precipitation, along with its lagged behavior concerning these variables. For this purpose, a Fourier spectral density analysis was implemented beforehand to know the maximum signal variation (spectral density) and acknowledge any temporal trend in the variance for each variable [28]. Estimated spectral density values were further used to calculate the cross-correlation coefficients that measure the correlation of periodic variations in total litterfall productivity vs temperature and precipitation. The time between variables with the highest correlation coefficient represents the response’s temporal dynamics (lag) [29].

Additionally, the anomalies presence was identified for each time series. The definition of anomalies in the context of this work matches two attributes: daily refers to extreme values occurring without modifying the mean response; e.g., tropical cyclones associated with daily precipitation. On the contrary, monthly anomalies are extreme values that modify the monthly mean.

Detection of the monthly anomalies was carried out at two temporal scales. The method consisted of removing seasonality and trend of observed data so that, the anomalies and their confidence intervals were detected on the residual values, using the interquartile range (IQR) of +/- 25 the median [30] and a 1-α = 0.95. The programming language R version 3.6.3 [31] was used to undertake this analysis while the functions: tk_anomaly_diagnostics from the timetk library [32] and time_decompose, anomalize, and time_recompose come from the anomalize library [33].

Systematic comparison of every anomaly with the most frequent range of values for a given variable along the entire data period (1961 to 2020) allows defining whether the anomaly was either a high or a low value for the month of each variable.

Finally, the potential relationship between litterfall with average temperature and total precipitation was analyzed, in joint with their anomalies. General additive model (GAM) further estimated the trend of total litterfall concerning total precipitation and mean temperature. A Poisson distribution was used to estimate the GAM model error distribution. GAM figures show the predicted values for litterfall with total precipitation and mean temperature. In both cases, the trend line was adjusted with cubic smoother [34]. GAM construction was achieved using the mgcv library [35]. Anomalies of litterfall with total precipitation and mean temperature were plotted with scatterplot of the ggplot2 library [36].

Two possible anomalies pathways were observed for each climatic variable, high and low (Table 1), as long as the recorded values were either higher or lower than expected.

**Table 1:** Description of the directionality of the anomaly for each climatic variable.

Climatic variable	High anomaly	Low anomaly
Maximum temperature	Abnormally high values for maximum temperature	Abnormally low values for maximum temperature
Minimum temperature	Abnormally high values for minimum temperature	Abnormally low values for minimum temperature
Mean temperature	Abnormally high values for mean temperature	Abnormally low values for mean temperature
Total precipitation	Abnormally high values for total precipitation	Abnormally low values for total precipitation
Total litterfall	Abnormally high values for total litterfall	Abnormally low values for total litterfall

## Results

**Fig 2:**
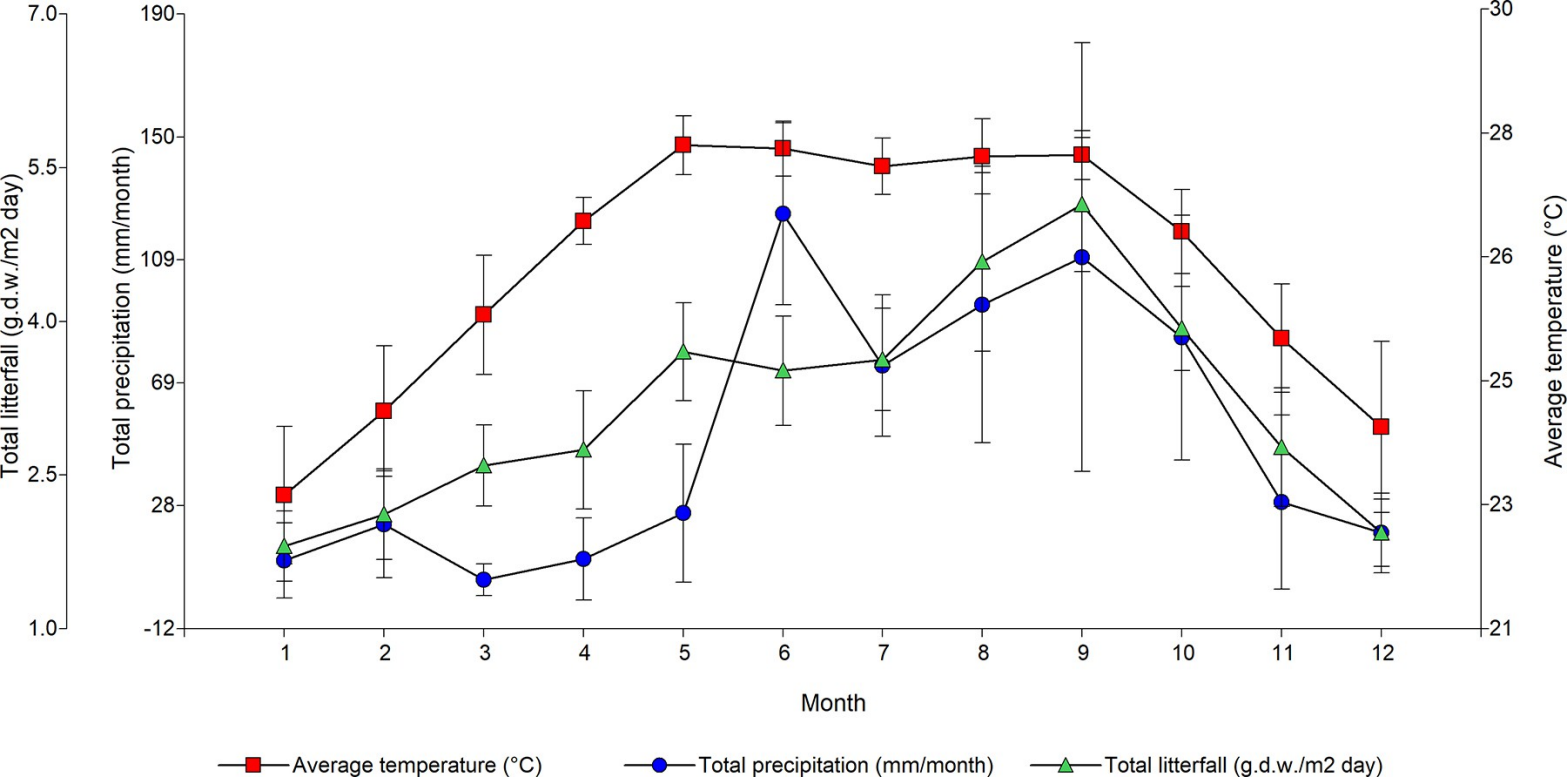
Monthly confidence interval at the 95% of mean temperature (°C), total litterfall (g.d.w.m^-2^ day^-1^) and total precipitation (mm month^-1^), from 1999 to 2010.

The monthly variability (e.g., mid-term variation level) indicates a pattern of maximum total litterfall production occurring between August and October, concomitant high precipitation and average temperatures (Fig 2).

**Fig 3:**
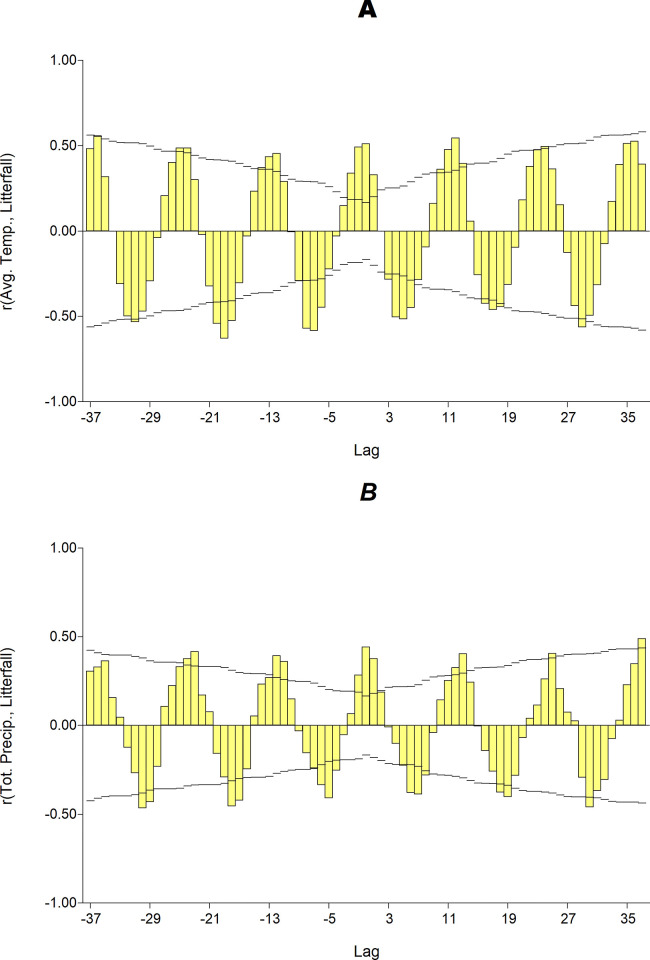
Cross-correlation graph: A) monthly mean temperature vs. litterfall and B) monthly total precipitation vs. litterfall.

A synchrony between litterfall production, mean temperature and litterfall and total precipitation is evident, with an estimated 2-months lag maximum for the litterfall response (Fig 3). In agreement with this time-varying behavior, 2 months before litterfall anomalies were considered to relate climatic anomalies with litterfall.

A total of 10 cyclonic events affected the Celestun Reserve (within the designated buffer) between 1961 and 2020, although any of them fell within the hurricane category. As aforementioned, other hurricanes impacting farther from the buffer were still considered. The tropical cyclones detected by the SMN [27] accounting up to 24, brought about profuse rain associated with every event nearby Celestun and adjacent sites. Thus, 99 daily precipitation anomalies (mm day^-1^) are identified between 1961 and 2020, while 15 are associated with 11 tropical cyclones (S1 Table).

**Fig 4:**
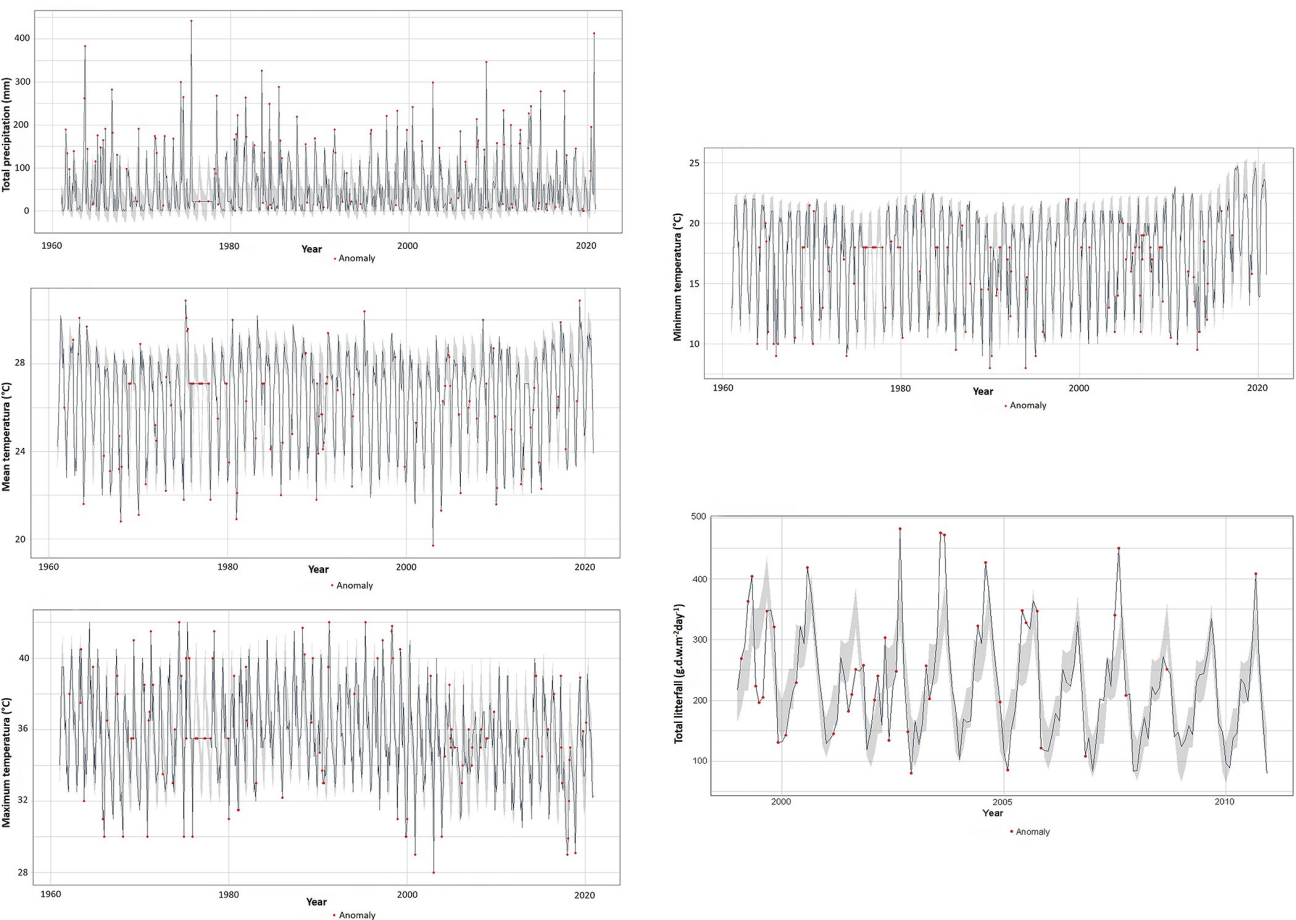
Anomalies: A) monthly total precipitation (mm month^-1^), B) monthly mean temperature (°C), C) monthly maximum temperature (°C), D) monthly minimum temperature (°C), and E) total litterfall (g.d.w. m^-2^ day^-1^). Red lines delimit the period of coincidence between climatic variables and litterfall. Shadow gray is the confidence interval of 95%.

Regarding monthly anomalies encompassing 1999–2010, 19 anomalies were detected for total monthly precipitation (mm month^-1^, Fig 4A), 20 for mean monthly temperature (°C, Fig 4B), 24 for maximum temperature (°C, Fig 4C), 27 for minimum temperature (°C, Fig 4D), and 43 for total litterfall (g.d.w. m^-2^ day^-1^, Fig 4E). In general, under the scope of the whole data series, increases both in mean and minimum temperatures are observed for the last 5 years. The maximum temperature depicted an overall downslope trend in the last 20 years. The litterfall production mirrored this downward pattern.

Considering the lag detected, 53.5% of the litterfall anomalies are related to one or more anomalies of either monthly or daily climatic variables (Table 2). Regarding these related anomalies, 60.9% are linked to one variable exclusively, and 43.5% are related to precipitation, either as a single variable or in joint with others.

**Table 2:** Litterfall anomalies related to monthly climatic anomalies and/or daily (cyclonic events), considering a maximum lag of 2 months (previous) and showing the direction of each anomaly (MxT = maximum temperature, MnT = minimum temperature, MeT = mean temperature, P = total precipitation, Ce = cyclonic event).

Date	Total litterfall (g.d.w. m-^2^ day^-1^)	Related climatic variables	Anomaly direction				
			Litterfall	MxT	MnT	MeT	P
Jun-1999	223.03	Mxt	Low	High	-	-	-
Dec-1999	130.47	P, Mxt, Met	Low	Low	-	Low	Low
Feb-2000	142.86	Mxt	Low	Low	-	-	-
May-2000	229.57	Met	Low	-	High	-	-
Aug-2000	418.30	P	High	-	-	-	High
Mar-2001	144.86	Mnt, Met	Low	-	High	High	-
Aug-2001	209.47	P	Low	-	-	-	High
Nov-2002	148.55	P, Mxt	Low	High	-	-	High
May-2003	202.47	Mnt	Low	-	Low	-	-
Aug-2003	475.27	P	High	-	-	-	High
Sep-2002	472.41	Ce	High	-	-	-	-
Jun-2004	322.93	Mxt, Mnt, Met	High	Low	Low	Low	-
Aug-2004	426.59	P	High	-	-	-	Low
Dec-2004	197.19	Mxt, Mnt, Met	High	High	High	High	-
Feb-2005	85.70	Mxt, Mnt, Met	Low	High	High	High	-
Jul-2005	327.53	Mxt	High	Low	-	-	-
Oct-2005	346.73	Ce	High	-	-	-	-
Nov-2005	121.41	P, Mnt	Low	-	Low	-	Low
Nov-2006	107.79	Mnt	Low	-	Low	-	-
Jul-2007	340.49	Mxt	High	Low	-	-	-
Oct-2007	207.85	P	Low	-	-	-	High
Sep-2008	251.26	P, Met	Low	-	-	High	Low
Sep-2010	408.63	P	High	-	-	-	High

**Fig 5:**
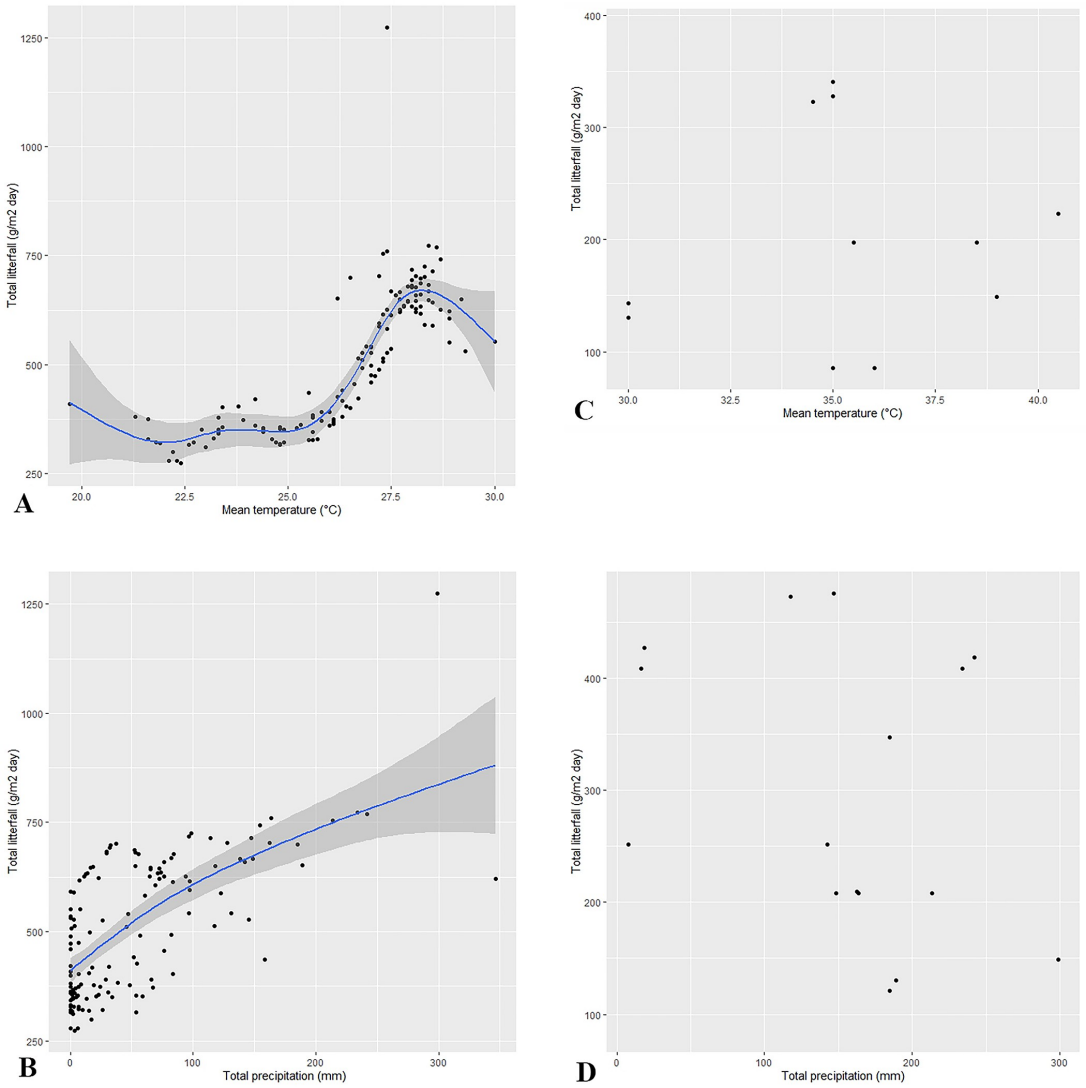
A) Total litterfall production trend (g.d.w. m^-2^ day^-1^) concerning monthly mean temperature (°C); B) trend of total litterfall production (g.d.w. m^-2^ day^-1^) concerning total monthly precipitation (mm); C) total litterfall anomalies (g.d.w. m^-2^ day^-1^) concerning anomalies of mean monthly temperature (°C); D) total litterfall anomalies (g.d.w. m^-2^ day^-1^) concerning anomalies of total monthly precipitation (mm). A and B depict GAM analysis, while C and D are scatterplots.

As to the trend detected, maximum litterfall production (August to October) occurs both at high temperatures and precipitation (Fig 5A and 5B) (Deviance explained = 50%, p = 2e-16). However, litterfall anomalies reflected not a defined response pattern regarding individual climatic variables anomalies, mean temperature r = 0.47, p = 0.28 and total precipitation r = -0.18, p = 0.53 (Fig 5C and 5D); that is, the response can be highly uncertain (Table 2).

## Discussion

Concurrent peak litterfall production with high temperatures and precipitation in Celestun lagoon during June and October (Fig 2) agrees with synchronic patterns reported in mangroves undergoing intense winds as well as superior temperature and rainfall, in joint with low salinity [37–39]. Yet, some delay in biotic responses upon climatic changes might be expected, temporarily masking the potential relationship between plant traits and the environmental setting [40]. The lack of a promptly manifestation in mangrove structure may reflect physiological processes requiring a response time, such as flowering triggered by water level fluctuations or porewater salinity, sea temperature, and air [41].

This study found a sort of ~ 2 months latency in the litterfall production response to climatic variables (Fig 3). Sánchez-Núñez and Mancera-Pineda [42] reported a time-lag response of physiological processes in mangroves delaying the onset of flowering nearly one month after water balance cues. In addition, Songsom et al. [41] and Pastor-Guzman et al. [39] found a variable lag (two-three month) of greenness (Normalized Difference Vegetation Index) coupled to precipitation.

The combination of high temperatures and high precipitation enhancing litterfall production may stem from the fact that warm season co-vary with day-length (more hours of sunlight), an overall condition favoring photosynthesis rates. In addition, maximum precipitation drives high recharge of the karstified Yucatan aquifer system and consequently, a larger ground freshwater flow controls the litterfall production [43]. Moreover, events accompanied by abundant precipitation have a diluting effect over the salinity of water bodies bounded by mangroves and within their soils, leading both to interstitial salinity decreasing and high nutrients availability, boosting the forest productivity [37].

Mounting evidence suggests extreme values of main driving factors (e.g., temperature and precipitation) play a strong control over mangrove productivity [43]. The Yucatan Peninsula is along the corridor of Atlantic hurricanes (extreme events of high intensity and short duration) and the Celestun area categorized with medium incidence of both tropical depressions and storms [37]. As early mentioned, for the period under study cyclones into the hurricane category did not directly affect nearby the study site, but rather atypical daily precipitations were imparted by hurricanes and tropical storms traversing further away (S1 Table). This finding shows the potential influence that heavy rainfall and strong winds reaching up to 450 km and up to 1000 km from the vortex of a tropical storm may represent for the forest structure [44].

The importance of extreme values, either high or low anomalies (Table 1), is reflected in the fact that above 53% of the litterfall production anomalies is owed to climatic variables anomalies. Climatic anomalies stress large, exposed mangroves areas. Besides the seasonal cycle underlying a joint climatic variables and litterfall production behavior (Fig 3), the set of anomalies detected constitutes atypical records for temperature, precipitation, cyclonic events and anomalous litterfall production as well.

Three cyclonic events made landfall in Celestun between 1999 and 2010 (Isidore in 2002; Stan and Wilma during 2005) sustaining litterfall anomalies at a daily and/or monthly level (Table 2), even though Isidore was the only reaching a hurricane category. No precipitation anomaly was detected at a monthly level for the other two events, but at a daily level nearby Celestun. On the other hand, Stan crossed directly through the Celestun area, yet portrayed a lessened rainfall signal than the other two events. Since intense winds associated with hurricanes cause defoliation, branch rupture, uprooting and tree fall [9, 10, 45], they play a major role in the magnitude of litterfall accumulated over the forest floor.

Regarding temperature variability, recently minimum and mean records exhibit a steadily increase (Fig 4). Contrastingly, the litterfall data time frame does not coincide with this pattern, preventing to associate temperature as a driver of litterfall production. As to the potential damage that vegetation undergo upon extremely low temperatures they include sudden loss of leaves, xylem embolism, reduction of branches and stems, absence of leaf production, and tree mortality in the most extreme cases. On the other hand, high temperatures undermine photosynthesis and increase evaporation, resulting in hypersalinity that constrains productivity [6, 19, 46].

This study provides insightful patterns of litterfall dynamics in response to precipitation and temperature. The foremost behavior highlights surplus litterfall production under high temperature (Fig 5A) and precipitation (Fig 5B). Regarding the anomalies (Table 2), a unique pattern of litterfall production relative to temperature or precipitation anomalies was lacking (Fig 5C and 5D). As far as this study concerns, no available publication develops an in-depth analysis of litterfall production anomalies. Still, some studies detected yearly anomalies ascribed to mid-term changes in the climatic regime–but not attribution to specific events [38, 47].

Gomes et al. [19] found an overall forest productivity decline in mangroves experiencing water balance shortage and excessive tree evapotranspiration. This seasonal-scale response led up to 30-fold higher litterfall production in undisturbed vegetation areas relative to those enduring a hydrologic impairment (reflecting hydric deficit and drought severity exceeding specific tolerance for dominant mangroves inhabiting a given location rather than solely the effect of thermally-driven stress). The breadth of these studies may have overlooked time-dependent structures in environmental variables and circumvent the specific trajectories that litterfall may follow in the aftermath of extreme values of key climatic variables. The elusiveness of such dynamic responses in mangrove structure encourages to pursue a thoroughly impact assessment by monitoring. This approach will enable foresee changes in litterfall associated with forthcoming global climate varying patterns and local developmental trends.

Prevailing climate change projections point out sustained pressing over region-wise intensification of the water cycle, leading to an enhanced frequency, magnitude, and precipitation volume provoked by tropical cyclones [5]. The overall hurricanes effect may be viewed in terms of either a single event or, as a disturbance regime over the ecosystems [11]. The Celestun Lagoon is a extensive transitional vegetation area where recurrent hydrometeorological events have the potential to influence not only the landscape structure along the coastal wetlands embodied by mangrove ecotypes, but also that of ecological processes as litterfall production, which varying behavior and relative production among constituents can smooth out potentially sharp responses of litterfall [11].

Besides individual-scale mechanical strength of mangrove trees against stormy winds, the intact-to-disturbed patch-size ratio may result in a configuration alternating residual vegetation surviving past disturbances with weakened areas across the landscape [48]. Such a mosaic combining biotic and abiotic instabilities may optimize the connectivity between impervious seeds and propagules bank with open spaces ready for colonization [11]. Hence, litterfall may also play an important role in harboring propagules dispersed under stormy events in the forests floor.

The study of extreme compound events is an incipient and rapidly advancing area of research [47]. The insights of the current study contributes to fulfil this need, providing a nuanced understanding of how extreme events elicit litterfall variability in mangroves. For instance, the extent to which litterfall is associated with wind-induced defoliation under extreme events depicts an ample variability [49]. This trait alters the potential of litterfall to modulate further ecological recovery trajectories, as different constitutes (e.g., leaves versus varying-size twigs or, flower/fruits) ensue particular decaying rates and properties. Some of them readily accruing in the forest floor to the expenses of a sluggish decomposition (i.e., woody debris) that heavily influences soil biogeochemistry, implying cyclone legacies at the ecosystem level, while others undergo relative quick sedimentary processing (i.e., leaves) and thus, are prone to follow metabolic pathways and materials recycling soon after the hydrometeorological event [11].

Finally, the potential greenhouse gas (GHG) offset through CO_2_ sequestration underscores the relevance to focus in the role mangrove forests services may play as a nature-based solutions. One important pathway within the mangrove ecosystem contributing to the C-cycle capture is the litterfall turnover rate, plus recycling of other components (trunks, branches) detached during stormy events. The organic C sequestration and accumulation in soils is stimulated by prevailing anaerobic conditions due to long-lasting flooding episodes caused by extreme precipitation during hydrometeorologic events. It is likely a temporal synchronization operates between precipitation seasonality and a decelerating litterfall decay. The former contributes to increasing flooding time, a driving factor underlying a highly efficient organic carbon storing capacity within these tropical ecosystems. Climate anomalies could then be pivotal to CO_2_ capture and sequestration pulses, which illustrates why it is important to ensure healthy mangrove ecosystems so they are still able contributing to GHG mitigation.

## Conclusions

Some of the litterfall production anomalies observed in mangroves of Celestun can be ascribed to daily anomalies, associated with cyclonic events (both air temperature and precipitation extremes). However, these disturbances and the damaged exerted over the mangrove forest enduring these hydrometeorological events do not consistently resemble the oscillations observed in the monthly values of climatic variables.

Monthly anomalous behavior of major drivers occurs in two ways, either abnormally high or low. The abnormalities direction set further at least two types of anomalies for each climatic variable, creating a wide range of possible combinations of anomalies and diverse litterfall responses, avoiding detection of a specific pattern. On the other way, the high proportion of litterfall anomalies related to climatic ones within the detected lag time, and the response of litterfall production to variables such as average temperature and total precipitation, indicates that climatic variables are major driving factors influencing mangrove productivity dynamics.

## Supporting information

S1 Table Tropical cyclones associated with daily precipitation anomalies (mm day-1, N/I = no information), according to their category of the Saffir-Simpson scale and the speed at the closest site to Celestun (TD = tropical depression, TS = tropical storm, H1 = hurricane category 1, H2 = hurricane category 2, H3 = hurricane category 3, H4 = hurricane category 4). (DOCX)

## References

[1] Rao M.P., Davi N.K., Magney T.S. et al. Approaching a thermal tipping point in the Eurasian boreal forest at its southern margin. Commun Earth Environ 4, 247 (2023). 10.1038/s43247-023-00910-6

[2] Tang H., Nolte S., Jensen K., Rich R., Mittmann-Goetsch J., and Mueller P. 2023. Warming accelerates belowground litter turnover in salt marshes–insights from a Tea Bag Index study. Biogeosciences, 20: 1925–1935. 10.5194/bg-20-1925-2023.

[3] Thompson V., Mitchell D., Hegerl G.C. et al. The most at-risk regions in the world for high-impact heatwaves. Nat Commun 14, 2152 (2023). doi: 10.1038/s41467-023-37554-1 37185667 PMC10130074

[4] Hagger V.; Worthington T.A.; Lovelock C.E.; Adame M.F.; Amano T.; Brown B.M.; et al. 2022. Drivers of global mangrove loss and gain in social-ecological systems. Nature Communications, 13(1): 6373. doi: 10.1038/s41467-022-33962-x 36289201 PMC9606261

[5] IPCC. 2023: Synthesis report of the IPCC sixth assessment report (AR6). Climate change 2023. Longer Report.

[6] Xiong L.; Lagomasino D.; Charles S.P.; Castañeda-Moya E.; Cook B.D.; Redwine J.; et al. 2022. Quantifying mangrove canopy regrowth and recovery after Hurricane Irma with large-scale repeat airborne lidar in the Florida Everglades. International Journal of Applied Earth Observation and Geoinformation, 114: 103031. 10.1016/j.jag.2022.103031

[7] Smith T.J. III, Anderson G.H., Balentine K., Tiling G., Ward G.A., and Whelan K.R.T. 2009. Cumulative impacts of hurricanes on Florida mangrove ecosystems: sediment, deposition, storm surges and vegetation. BioOne, 29: 24–34, doi: 10.1672/08-40.1

[8] Taillie P. J., Roman-Cuesta R., Lagomasino D., Cifuentes-Jara M., Fatoyinbo T., Ott L. E. et al. 2020. Widespread mangrove damage resulting from the 2017 Atlantic mega hurricane season. Environ. Res. Lett., 15: 064010, doi: 10.1088/1748-9326/ab82cf

[9] Imbert D. 2018. Hurricane disturbance and forest dynamics in east Caribbean mangroves. Ecosphere 9(7): e02231, doi: 10.1002/ecs2.2231

[10] Fickert T. 2018. Better Resilient than Resistant-Regeneration Dynamics of Storm-Disturbed Mangrove Forests on the Bay Island of Guanaja (Honduras) during the First Two Decades after Hurricane Mitch (October 1998). Diversity, 10, 8: doi: 10.3390/d10010008

[11] Krauss Ken; Osland Michael. 2020. Tropical cyclones and the organization of mangrove forests: A review. Annals of botany, 125: 213–234. doi: 10.1093/aob/mcz161 31603463 PMC7442392

[12] Asbridge Emma; Lucas Richard; Rogers Kerrylee; Accad Arnon. 2018. The extent of mangrove change and potential for recovery following severe Tropical Cyclone Yasi, Hinchinbrook Island, Queensland, Australia. Ecology and Evolution, 8(21): 10416–10434. doi: 10.1002/ece3.4485 30464815 PMC6238134

[13] Hill, Jack W.; Bennion Vicki; Lovelock, Catherine E 2024. Mangrove tree strength estimated with field experiments. Ecological Engineering, 203: 107259. 10.1016/j.ecoleng.2024.107259

[14] Dominguez C.; Done J.M.; Bruyère C.L. Future Changes in Tropical Cyclone and EasterlyWave Characteristics over Tropical North America. Oceans 2021, 2, 429–447. 10.3390/oceans2020024

[15] Mchega I.S.S. and Rashid J.R. 2017. Mangrove litter production and seasonality of dominant species in Zanzibar, Tanzania. Journal of East African Natural History 106(1): 5–18.

[16] Zaldívar-Jiménez M.A., Herrera-Silveira J. A., Teutli-Hernández C., Comín F. A., Andrade J. L., Coronado-Molina C., et al. 2010. Conceptual Framework for mangrove restoration in the Yucatán Peninsula. Ecological Restoration, 28: 333–342, doi: 10.3368/er.28.3.333

[17] Zhao Xiaochen, Victor Rivera-Monroy Chunyan Li, Ivan Vargas Lopez, Robert V Rohli, Xue Zuo George et al. (2022). Temperature across Vegetation Canopy-Water-Soil Interfaces is Modulated by Hydroperiod and Extreme Weather in Coastal Wetlands. Front. Mar. Sci. 9:852901. doi: 10.3389/fmars.2022.852901

[18] Hopkinson C.; Wolanski E.; Brinson M.; Cahoon D.; Perillo G.; 2019. Coastal Wetlands: A Synthesis. In: Coastal Wetlands: An Integrated Ecosystem ApproachEdition: FirstChapter: Coastal Wetlands: A SynthesisPublisher: ElsevierEditors: Gerardo M.E. Perillo, Eric Wolanski, Donald R. Cahoon, Mark M. Brinson.

[19] Gomes L.E.; Vescovi L.C.; Bernardino A.F.; 2021. The collapse of mangrove litterfall production following a climate-related forest loss in Brazil. Marine Pollution Bulletin, 162: 111910. doi: 10.1016/j.marpolbul.2020.111910 33338926

[20] Herrera-Silveira J.A. 1994. Spatial heterogeneity and seasonal patterns in a tropical coastal lagoon. J. Coastal Res., 10: 738–746.

[21] Prado-Roque S. A. 2008. Estrategia preliminar para la aplicación de la política de gestión del agua por cuenca en la Región XII, Península de Yucatán. Gerencia Regional de la Península de Yucatán de la Comisión Nacional del Agua. México.

[22] Kjerfve B. 1994. Coastal lagoons. In: Kjerfve B. (Ed.), Coastal lagoons processes. Elsevier Science Publishers, New York, 1–8 p.

[23] Caamal-Sosa J. P, Zaldívar A., Adame -Vivanco F., Teutli C., Andueza M. T., Pérez R. et al. 2012. Almacenes de carbono en diferentes tipos ecológicos de manglares en un escenario cárstico. En Paz F. Cuevas y R.(editores). Estado Actual del Conocimiento del Ciclo del Carbono y sus Interacciones en México: Síntesis a 2011. Texcoco, Estado de México, México. ISBN 978-607-715-085-5.

[24] Brow M. 1984. Mangrove litter production and dynamics. 231–237 p. In: Snedaker S.C. and Snedaker J.G. (eds.). The mangrove ecosystem: Research methods. Monographs on oceanographic methodology 8. UNESCO/SCOR. UK. 251 p.

[25] CONAGUA. 2021. Resúmenes Mensuales de Temperaturas y lluvia. Comisión Nacional del Agua‐Servicio Meteorológico Nacional. En https://smn.conagua.gob.mx/es/climatologia/temperaturas-y-lluvias/resumenes-mensuales-de-temperaturas-y-lluvias

[26] Knapp K. R., Diamond H. J., Kossin J. P., Kruk M. C., Schreck C. J., 2018: International Best Track Archive for Climate Stewardship (IBTrACS) Project, Version 4. [North Atlantic]. NOAA National Centers for Environmental Information. doi: 10.25921/82ty-9e16[02/20/22]

[27] SMN. 2021. Lluvias asociadas a ciclones tropicales. Subgerencia de Pronóstico a Mediano y Largo Plazo, Subgerencia de Monitoreo Atmosférico Ambiental. Comisión Nacional del Agua. Consulta realizada en octubre 2021. En https://smn.conagua.gob.mx/es/ciclones-tropicales/lluvias-asociadas-a-ciclones-tropicales

[28] Scharlemann J.P.W., Benz D., Hay S. I., Purse B. V., Tatem A. J., Wint G. R. W., et al. 2008. Global data for ecology and epidemiology: a novel algorithm for temporal Fourier processing MODIS data. PLoS ONE 3, e1408, doi: 10.1371/journal.pone.0001408 18183289 PMC2171368

[29] Olden J. D. and Neff B. D. 2001. Cross-correlation bias in lag analysis of aquatic time series. Mar. Biol., 138: 1063–1070, doi: 10.1007/s002270000517

[30] Cleveland R.B., Cleveland W.S., McRae J.E., and Terpenning I. 1993. STL: A Seasonal-Trend Decomposition Procedure Based on Loess. Journal of Official Statistics, 6:3–73.

[31] R Core Team. 2020. R: A language and environment for statistical computing. R Foundation for Statistical Computing, Vienna, Austria. URL https://www.R-project.org/

[32] Dancho M. and Davis Vaughan. 2021. Timetk: A Tool Kit for Working with Time Series in R. R package version 2.6.1. https://CRAN.R-project.org/package=timetk

[33] Dancho M. and Vaughan D. 2020. Anomalize: Tidy Anomaly Detection. R package version 0.2.2. https://CRAN.R-project.org/package=anomalize

[34] Wood S. N., Pya N. and Saefken B. 2016. Smoothing parameter and model selection for general smooth models (with discussion). Journal of the American Statistical Association 111:1548–1575, doi: 10.1080/01621459.2016.1180986

[35] Wood S. N. 2017. Generalized Additive Models: An Introduction with R (2nd edition). Chapman and Hall/CRC. ISBN: 9781498728331.

[36] Wickham H. 2016. ggplot2: Elegant Graphics for Data Analysis. Springer-Verlag New York. USA.

[37] Adame M. F., Zaldívar-Jiménez A., Teutli C., Caamal J. P., Andueza M. T., López-Adame H., et al. 2013. Drivers of Mangrove Litterfall within a Karstic Region Affected by Frequent Hurricanes. Biotropica, 45: 147–154, doi: 10.1111/btp.12000

[38] Agraz-Hernández C. M., Chan Keb C. A., Iriarte-Vivar S., Posada-Venegas G., Vega-Serratos B., and Osti-Sáenz J. 2015. Phenological variation of *Rhizophora mangle* and ground water chemistry associated to changes of the precipitation. Hidrobiológica, 25: 49–61, ISSN 0188-8897.

[39] Pastor-Guzman J., Dash J., and Atkinson P. M. 2018. Remote sensing of mangrove forest phenology and its environmental drive. Remote Sensing of Environment, 205: 71–84, doi: 10.1016/j.rse.2017.11.009

[40] Anderegg LDL. 2023. Why can’t we predict traits from the environment. New Phytologist, 237: 1998–2004. doi: 10.1111/nph.18586 36308517

[41] Songsom V., Koedsin W., Ritchie R. J., and Huete A. 2019. Mangrove Phenology and Environmental Drivers Derived from Remote Sensing in Southern Thailand. Remote Sens., 11(8), 955, doi: 10.3390/rs11080955

[42] Sánchez-Núñez D. A. and Mancera-Pineda J. E. 2011. Flowering patterns in three neotropical mangrove species: Evidence from a Caribbean island. Aquatic Botany, 94: 177–182, doi: 10.1016/j.aquabot.2011.02.005

[43] Bernini E. and Rezende C. E. 2010. Litterfall in a mangrove in Southeast Brazil. Pan-American Journal of Aquatic Sciences, 5:508–51.

[44] Frank W. M. 1977. The Structure and Energetics of the Tropical Cyclone I. Storm Structure. Monthly Weather Review, 105: 1119–1135, doi: 10.1175/1520-0493(1977)105

[45] Ebi K. L., Vanos J., Baldwin J. W., Bell J. E., Hondula D. M., Errett N. A., et al K. 2021. Extreme Weather and Climate Change: Population Health and Health System Implications. Annu. Rev. Public Health, 42: 293–315, doi: 10.1146/annurev-publhealth-012420-105026 33406378 PMC9013542

[46] Ward R. D., Friess D. A., Day R. H., and Mackenzie R. A. 2016. Impacts of climate change on mangrove ecosystems: a region by region overview. Ecosystem Health and Sustainability, 2:4, doi: 10.1002/ehs2.1211

[47] Lovelock C. E., Krauss K. W., Osland M. J., Reef R., and Ball M. C. 2016. The Physiology of Mangrove Trees with Changing Climate. In Goldstein G. and Santiago L. S., Tropical Tree Physiology Adaptations and Responses in a Changing Environment. Springer International Publishing Switzerland. Switzerland. Pp. 149–179, doi: 10.1007/978-3-319-27422-5_7

[48] Turner, Monica; Gardner, Robert; 2015. Landscape ecology in theory and practice: Pattern and process, second edition. 482 pp. SN—978-1-4939-2793-7. doi: 10.1007/978-1-4939-2794-4

[49] Krauss KW, McKee KL, Lovelock CE, et al. 2014. How mangrove forests adjust to rising sea level. New Phytologist 202: 19–34 doi: 10.1111/nph.12605 24251960

