# Supplementary material for: Relationship of litterfall anomalies with climatic anomalies in a mangrove swamp of the Yucatan Peninsula, Mexico
Source: PLoS One. 2024 Aug 28;19(8):e0307376. doi: 10.1371/journal.pone.0307376 (PMC11356426; doi:10.1371/journal.pone.0307376)
Supplement: S1 Table — (DOCX) [file pone.0307376.s001.docx]

**Supporting information**

**S1 Table 1. Tropical cyclones associated with daily precipitation anomalies (mm day-1, N/I = no information), according to their category of the Saffir-Simpson scale and the speed at the closest site to Celestun (TD=tropical depression, TS=tropical storm, H1=hurricane category 1, H2=hurricane category 2, H3=hurricane category 3, H4=hurricane category 4).**

| **Year** | **Month** | **Name** | **Saffir-Simpson**  **category** | **Maximum sustained wind speed (km/h)** | **Accumulated precipitation during cyclone (mm) in Celestún** |
| --- | --- | --- | --- | --- | --- |
| 1966 | October | Inez | H4 | 213 | 100-125 |
| 1973 | August | Brenda* | TS | 83 | 100-125 |
| 1974 | June | DT1 | TD | N/I | 200-300 |
| 1974 | September | Carmen | TS | 96 | 150-200 |
| 1988 | September | Gilbert | H2 | 176 | 150-200 |
| 1990 | August | Diana* | TS | 83 | 70-100 |
| 1995 | September | Opal* | TS | 83 | 70-100 |
| 2002 | September | Isidore | H1 | 130 | 300-400 |
| 2005 | October | Stan* | TD | 56 | 100-125 |
| 2005 | October | Wilma | H3 | 193 | 100-125 |
| 2020 | June | Cristobal | TS | 65 | 150-200 |
| 2020 | October | Gamma | H1 | 142 | 150-200 |
| 2020 | October | Zeta | TS | 106 | 20-35 |

* Cyclones outside the 40 km buffer.
